# Physical, Cognitive, and Psychosocial Predictors of Functional Disability and Health-Related Quality of Life in Adolescents with Neurofibromatosis-1

**DOI:** 10.1155/2012/975364

**Published:** 2012-09-26

**Authors:** Molly M. Garwood, Jessica M. Bernacki, Kathi M. Fine, Keri R. Hainsworth, W. Hobart Davies, Bonita P. Klein-Tasman

**Affiliations:** ^1^Children's Hospital of Wisconsin Community Services, 620 S. 76th Street, Suite 120, Milwaukee, WI 53214, USA; ^2^Department of Psychology, University of Wisconsin-Milwaukee, P.O. Box 413, 2200 E. Kenwood Boulevard, Milwaukee, WI 53226, USA; ^3^Department of Anesthesiology, Medical College of Wisconsin, 9000 Wisconsin Avenue, Milwaukee, WI 53226, USA

## Abstract

*Objective*. To examine physical, cognitive, and social-emotional predictors of quality of life (HRQOL) and functional disability (FD) in adolescents diagnosed with Neurofibromatosis-1. *Methods*. Participants were twenty-seven adolescents with a diagnosis of NF-1 who were recruited through an NF-1 specialty clinic at a large Midwestern children's hospital. Measurements of the adolescents' cognitive functioning, pain, FD, HRQOL, and social and emotional functioning were obtained with corresponding parent measures. *Results*. Emotional functioning significantly predicted youth-reported and parent-reported HRQOL, whereas days of pain significantly predicted youth-reported FD. *Conclusions*. NF-1 is a complex disease. Measurements of the overall impact of the disease tap into different aspects of the effects of NF-1 on daily life. Global outcomes such as HRQOL appear to be influenced especially by emotional functioning, whereas outcomes such as FD appear to be influenced by the physical/organic aspects of NF-1.

## 1. Introduction

Neurofibromatosis-1 (NF-1) is one of the most common autosomal dominant disorders affecting the nervous system [1] and occurs in approximately 1 in 4000 individuals [2]. NF-1 occurs equally in all racial and ethnic groups and is best characterized by the presence of skin-pigment abnormalities called café-au-lait spots and the development of benign tumors on or underneath the skin [1]. NF-1 is the result of a mutation on chromosome 17 that is either inherited by an affected parent or occurs as a random genetic mutation during development [1]. While the genetic origin is known, NF-1 is generally diagnosed based on the presence of physical symptoms rather than genetic testing [3].

The physical symptoms of NF-1 can greatly alter quality of life. For example, plexiform neurofibromas may impact movement and focal scoliosis often requires corrective surgery. Pain has been examined as a possible consequence of NF-1 and has been linked with impaired quality of life in this population. Chrusciel and colleagues [4] reported that pain was common in children with NF-1, with 77% of their sample reporting pain associated with lower self-reported quality of life. In adults with NF-1, the literature suggests that headache pain is the predominant form of pain experienced by youth with NF-1; however, the prevalence rates are mixed. Créange and colleagues [5] found that headaches were reported by 18% of patients and it was the predominant complaint in 11%. Clementi et al. [6] reported rates of headaches around 30%, while DiMario and Langshur [7] reported significantly higher rates of 61% that interfered with daily activities.

In addition to physical limitations, individuals with NF-1 experience difficulties cognitively, socially, and emotionally. Cognitive impairment is considered the most common neurologic complication of NF-1 in childhood [8]. Intellectual functioning is significantly lower in patients with NF-1 than in typically developing children; however, the degree of impairment is generally mild [9–11]. Additionally, previous research has shown incidence rates of learning disabilities (LDs) in children with NF-1 to range from 30% to 65% and incidence rates of attention deficit hyperactivity disorder (ADHD) to be as high as 50% [8, 11]. Difficulties in visuospatial skills, attention, executive functioning, and expressive and receptive language are also more common in youth with NF-1 [11, 12].

Children with NF-1 may also experience difficulties in their social and emotional functioning. Parent, teacher, peer, and self-reports of social functioning indicate that children with NF-1 experience significantly poorer social and emotional outcomes than their unaffected siblings and other normative comparison groups [13–15]. Children with NF-1 with greater attention difficulties also tend to show more social difficulties [13].

With such wide-ranging symptoms, it is reasonable to expect that adolescents with NF-1 may experience significant difficulties across life domains. Two common measures for the impact of disease on functioning are health-related quality of life (HRQOL) and functional disability (FD). Although related, these distinct constructs have disparate functions related to diagnosis, treatment, and the overall impact of disease [16]. HRQOL is defined as the impact of a disease or condition on physical health status, psychological and social functioning, and emotional well-being [17, 18]. In contrast, FD is the degree of impairment someone experiences due to an illness or medical condition [19]. The importance of examining these constructs independently has been demonstrated in the field of pediatric chronic pain [20, 21], with researchers stating that each adds unique outcome information.

Research about the general impact of NF-1 has been limited. French adults with NF-1 reported impaired HRQOL in both emotional and physical functioning [22]. Youth with NF-1 and their parents have reported significantly lower rates of motor, social, cognitive, and emotional functioning compared to healthy youth [23–28]. These parents also reported higher rates of internalizing and externalizing behavioral problems in their children compared to parents of healthy youth. Currently, no published studies have been reported on FD in youth with NF-1. In one study with adults with NF-1 [5], life-threatening complications were categorized according to their related FD, but degree of impairment was not specifically assessed. It is critical that we better understand factors related to functioning in youth with NF-1.

The goal of the current study is to independently examine the constructs of quality of life and functional disability in youth with NF-1. This research will provide a thorough description of the impact of NF-1 on physical, cognitive, and psychosocial functioning and seek to identify factors of this illness that predict difficulties in functioning that warrant further examination and intervention. With this information, healthcare professionals will be able to target specific domains when working with families having a child with NF-1 to help improve their overall functioning.

At the descriptive level, it is hypothesized that the adolescents with NF-1 in this sample will display a large degree of symptom variability and will report higher FD and lower HRQOL than expected based on a healthy normative sample. It is anticipated that aspects of physical, cognitive, and socioemotional functioning will significantly predict HRQOL and FD but that the specific predictors of HRQOL and FD will differ. It is expected that the more specific measure of FD will be predicted by cognitive and physical variables (i.e., cognitive functioning, days of pain), whereas the more global measure of HRQOL will be primarily influenced by social and emotional factors.

In summary, NF-1 is a disorder with a large degree of variability in clinical presentation and severity. Lower FD, physical symptoms, cognitive difficulties, and social and emotional difficulties are all sequelae of NF-1. Understanding the different ways to measure the overall impact of the NF-1 will allow for a fuller picture of the impact of disease on adolescents.

## 2. Methods

### 2.1. Participants

After getting approval from the hospital internal review board, adolescents with NF-1 and their families were recruited from a specialty clinic at a large Midwestern children's hospital. Adolescents all had a diagnosis of NF-1 made by a physician expert in the field, were between the ages of 12–18 years, lived within 120 miles of the hospital, and were English speakers. Thirty-seven families met these criteria and were approached to participate in the study during their annual appointment at the neurofibromatosis clinic. Two families refused participation and 10 families later cancelled their appointments due to scheduling problems. The final sample consisted of twenty-seven adolescents from twenty-five families.

### 2.2. Procedure

Families who expressed interest and met the eligibility criteria met with either a graduate or upper-level undergraduate student research assistant who described the research study and details of participation. If eligible and interested, parental consent and child assent were obtained. At this initial meeting, a pain diary was given for the adolescent to complete over the next 2 weeks and an appointment was scheduled for the assessment portion of the study. These assessments were completed in families' homes. Clinical psychology graduate students administered measures of cognitive functioning, functional disability, HRQOL, and social and emotional functioning. Parents were also asked to complete measures of their child's social and emotional functioning, HRQOL, and FD. If participants had questions while completing the measures (e.g, the meaning of a term or an item), assistance was provided (e.g., by explaining the item). All family members were given a $15 gift card to thank them for their participation.

### 2.3. Measures

The current study uses a portion of the assessment measures collected as part of a larger study examining psychosocial and neuropsychological functioning in youth with NF-1.

#### 2.3.1. Background Information

A background questionnaire was created for this study that asks parents to provide information about their child's school, medical, and mental health history, as well as sibling health; parents' ages, occupations, and education level; extended family medical history. This questionnaire included items asking parents to report whether or not they themselves had a diagnosis of NF-1.

#### 2.3.2. Medical Severity

Severity of disease was measured using a scale previously used by Reiter-Purtill et al. [29] in their study of parental distress and family functioning in children with NF-1. These authors created their scale by combining three previous scales of NF-1 severity including a scale of overall medical severity [30], a scale of the degree of visibility of NF-1 features [31], and a scale of neurological impairment [32]. For the current study, this combined scale was used and participants received a severity rating 1 (minimal) to 4 (severe) on three separate scales (i.e., general, appearance, and neurological). These ratings were completed by a clinical geneticist or physician in the NF-1 specialty clinic using medical chart review. An average severity score was calculated for use in the following analyses.

#### 2.3.3. Cognitive Functioning

The Kaufman Brief Intelligence Test-2nd Edition (KBIT-2) [33] was administered to evaluate cognitive functioning. The KBIT-2 is a screening measure of intelligence that assesses both verbal and nonverbal abilities. The KBIT-2 includes three subtests: verbal knowledge, riddles, and matrices used to calculate an IQ composite score (M = 100, SD = 15). While not a comprehensive measure of cognitive functioning, the KBIT-2 has demonstrated excellent reliability and internal consistency. This measure has also shown strong validity as a measure that is correlated with other intelligence tests as well as academic achievement measures [33].

#### 2.3.4. Social and Emotional Functioning

To assess social and emotional functioning, the adolescent Behavior Assessment System for Children—Second Edition (BASC-2) [34] was completed by both the child and at least one parent. This measure's multidimensional design and the use of multiple informants provide a comprehensive screen of children's behaviors. The Parent Rating Scale (PRS) assesses adaptive and problem behaviors in the home and community setting and the Self-Report-Adolescent (SRP-A) form provides information about the child's thoughts, feelings, and behavior. As a measure of overall social and emotional functioning, the BASC-2 self-report Emotional Symptoms Index and the parent-report Behavioral Symptoms Index were examined. These scales provide *t*-scores with a mean of 50 and standard deviation of 10. Scores of 60–69 indicate that the individual is “At Risk” of developing clinically significant problem and scores greater than 70 indicate “Clinically Significant” problems. Although these summary scales have different labels, the Emotional Symptoms Index and the Behavioral Symptoms Index both measure the construct of emotional functioning. These index scores will be used throughout the remainder of the paper to reflect youth self-reported and parent proxy-reported emotional functioning.

#### 2.3.5. Pain

Frequency and intensity of pain were assessed using daily pain diaries with a Visual Analog Scale (VAS) [35]. Pain diaries were completed at 3 time points (morning, midday, and before bed) each day, over a two-week period. Participants rated their *average pain *(0: “no pain,” 10: “worst pain possible”). This scale yielded a measure of how many days, over a 2-week period, the adolescent reported having pain. Previous research in youth with NF-1 has targeted the occurrence of headaches [4–7]. In the current study, the goal was to assess the frequency with which youth with NF-1 experience daily generalized pain and how this may be related to their current functioning.

#### 2.3.6. Health-Related Quality of Life

HRQOL was measured using the Pediatric Quality of Life Inventory [36]. The PedsQL is a 23-item multidimensional measure assessing children's and adolescent's perceptions of their quality of life during the past month. The child self-report form (8–12 years) and adolescent self-report form (13–18 years) were used. The PedsQL has demonstrated good reliability and validity across age groups [36]. Scores range from 0 to 100 with higher scores reflecting greater HRQOL. This measure has been shown to distinguish between healthy youth and youth with acute or chronic medical conditions and to be associated with morbidity and illness burden [36]. In one large study, healthy youth were shown to have average self-report total scores of 83.00 (SD = 14.79) compared to chronically ill youth (M = 77.19; SD = 15.53) and acutely ill youth (M = 78.70; SD = 14.04) [36]. This measure also distinguished between parent-reported quality of life in healthy youth (M = 87.61; SD = 12.33) compared to chronically ill youth (M= 74.22; SD = 18.40) and acutely ill youth (M = 80.42; SD = 15.26) [36].

#### 2.3.7. Functional Disability

Functional limitations for youth were assessed with the Functional Disability Inventory (FDI) [19]. The FDI is a one-dimensional scale rating perceptions of activity limitations because of physical health during the past two weeks. The FDI is a self-report inventory for children that measures perceived difficulty in performing a number of activities in the domains of school, home, recreation, and social interactions. It consists of 15 items rated on a 5-point scale (0 = no trouble to 4 = impossible) and yields total scores that can range from 0 to 60, with higher scores reflecting greater functional disability [37]. In addition to a child self-report form, a parent-proxy version exists and was also used in this study. In a sample of youth 5–17 years with chronic abdominal pain, average FDI scores were 11.25, with a range of 0–53 [19].

## 3. Results

### 3.1. Demographic and Background Characteristics

Twenty-six adolescents ages 12 to 18 years (54% female, M age = 13.65, SD = 1.88) were included in these analyses. One additional participant was recruited for this study but was dropped from the current analyses due to the fact that she was a significant outlier in terms of her level of cognitive functioning (IQ = 47). Twenty-five mothers and 13 fathers completed questionnaires. As not all youth had both parents' complete questionnaires, data from each adolescent's primary caregiver were used (25 mothers, 1 father). Eleven of the adolescents (42%) had a parent with NF-1 (seven mothers and four fathers) and six had an affected sibling (23%). Three youth (12%) were reported to have been held back a grade during their school career; 13 (50%) were reported to have an individualized education plan (IEP) or 504 plan; 11 (42%) were enrolled in special education classes. Seventy-five percent of parents reported attending postsecondary education. Participants were predominately Caucasian (88%), 8% identified themselves as African American, and 4% reported belonging to more than one racial group.

Adolescents with NF-1 fell within the minimal to moderate range for disease severity. Fifty percent fell in the minimal severity range indicating that that they had few NF-1 features and experienced no health complications. Twenty-seven percent fell in the mild severity range indicating they had symptoms such as mild hypertension or asymptomatic tumors. The remainder of the sample, 23%, fell in the moderate severity range. They presented with symptoms such as orthopedic complications or large or symptomatic plexiforms. Days of pain ranged from 0 to 14, with youth reporting on average 4.13 days of pain in the last two weeks. Scores on the KBIT ranged from 69 to 122 with a mean intelligence quotient (IQ) of 97.62. Sixty-two percent scored within the average range with 19% falling below average and 19% above average. On average, youth's ratings of quality of life were closer to youth with acute and chronic illnesses than healthy youth (see Table 1 for measure descriptives) [36]. Parents' ratings of their child's quality of life were lower than previously published parents' ratings of their chronically and acutely ill children. Overall, youth and parents reported low levels of functional disability although there was a considerable range with some reports of moderate impairment. On the BASC-2, both youth and parents reported emotional functioning scores within the average range. Twenty-three percent of parents rated their children as having emotional functioning scores within the “At Risk” range, and 19% within the “Clinically Significant” range. Eleven percent of youth reported symptoms falling within the “At Risk” range and one participant had a self-report score in the “Clinically Significant” range.

Kendall's tau bivariate correlations were used to examine the relationship between the primary variables (Table 2). This nonparametric statistic was used due to the small sample size. Disease severity was not significantly correlated with total days of pain, participant- and parent-reported HRQOL, FD, or emotional functioning. Significant correlations were observed between disease severity and cognitive functioning (*τ* = −.45, *P* < .01).

Gender and age differences were examined for HRQOL, FD, cognitive functioning, and emotional functioning. None of these variables were found to differ significantly by gender. Age was found to be significantly correlated with self-reported emotional functioning (*τ* = .40, *P* < .01) and parent-reported emotional functioning (*τ* = .39, *P* < .05).

### 3.2. Prediction of Health-Related Quality of Life and Functional Disability

#### 3.2.1. Health-Related Quality of Life

Hierarchical multiple regressions were used to examine the effects of age, pain, and emotional and cognitive functioning on child- and parent-reported HRQOL (see Table 3). Due to significant bivariate relations, age was entered as the first step in the regression analyses.

As shown in Table 3, 38% of the variance in child-reported HRQOL was explained by the model (*F* (4, 19) = 2.89,  *P* = .05). The first step of the analysis was not significant, with age accounting for only 14% of the variance. The second step, which incorporated pain and cognitive and emotional functioning, accounted for an additional 24% of the variance (*P* = .05). Emotional functioning was a significant individual predictor (*β* = −.47, *P* = .04), with higher scores on the BASC-2 (i.e., more clinically significant emotional symptoms) correlated with lower HRQOL.

The parent-report model was also significant (*F* (4, 19) = 7.93, *P* < .01) predicting 63% of the variance in HRQOL. Age, entered again as the first step, was not a significant predictor, accounting for only 8% of the variance. The second step, incorporating pain and cognitive and emotional functioning, was significant, accounting for an additional 55% of the variance (*P* < .01). Emotional functioning was again a significant individual predictor (*β* = −.75, *P* < .001), with higher scores on the BASC correlated with lower HRQOL.

#### 3.2.2. Functional Disability

Hierarchical multiple regressions were used to examine the effects of age, pain, and emotional and cognitive functioning on child- and parent-reported FD (see Table 4). Due to significant bivariate relations with age, age was entered as the first step in the regression analyses.

The child-report model was significant (*F* (4, 19) = 5.69, *P* < .01) predicting 55% of the variance in child-reported FD. Age, entered in the first step, was not a significant predictor of child-reported FD, accounting for only 2% of the variance. The second step which incorporated pain and emotional and cognitive functioning was significant, accounting for an additional 52% of the variance (*P* < .01). Total days of pain was a significant unique predictor (*β* = .61, *P* < .01), with greater days of pain correlated with greater FD. The parent-report model was not significant (*F* (4, 19) = 0.44, *P* = .78), accounting for only 9% of the variance in parent-reported FD.

## 4. Discussion

This study examined the effects of physical, cognitive, and emotional factors on the quality of life and functioning of adolescents with NF-1. A broad exploration of functioning was utilized due to the fact that NF-1 is a complex disease, with the potential to impact the lives of adolescents in myriad ways. To better understand the impact of NF-1, this study focused on two main outcomes: health-related quality of life (HRQOL) and functional disability (FD).

Consistent with study predictions, participants showed varied levels of functioning. Emotional functioning was found to be a significant predictor of HRQOL for both youth and parents but did not predict FD. Clinicians would do well to be aware that even children who do not exhibit decreased physical functioning could still be experiencing emotional difficulties that impact their quality of life. Measures of HRQOL in patients with NF1 can supplement measures of clinical severity and physical limitations to comprehensively assess the status of the patient and suggest treatment directions.

Also as expected, days of pain was a significant predictor of self-reported FD. It was not found to be a significant predictor of parent-reported FD. Consistent with findings from studies involving youth with chronic pain (see [19]), these results suggest that pain is an important indicator of functioning for adolescents with NF-1. It is possible that greater attention to pain management may mitigate pain intrusiveness and improve physical functioning. The findings should be taken with caution, however, as there were fewer self-reported days of pain in our sample than may have been expected from past literature that focused specifically on headache pain. It is possible that our measurement of pain was met with resistance from the adolescents who may have underreported their pain. Future research may consider adding parental report of youth pain or a shorter time-frame for pain reporting.

In contrast with our hypothesis and previous research findings, cognitive functioning did not predict either HRQOL or FD. Earlier studies have shown a relationship between aspects of cognitive functioning and neurological severity on social and emotional functioning [32, 38, 39]. Although the youth in our sample had similar IQ scores as reported by Martin et al. (2012), participants in the current study were much less likely to be in special education classes, which may reflect a discrepancy in the degree of impairment between these samples [39]. Barton and North (2004) also reported that attention deficit hyperactivity disorder (ADHD) was an important risk factor for difficulties in social and emotional functioning [13]. Rates of attention deficits were not obtained in the current study. It is possible that attentional difficulties were not as prevalent in the current study, contributing to a reduced relationship between emotional and cognitive functioning. Support for the need to assess a wider array of cognitive skills was demonstrated by Huijbregts and De Sonneville [38]. Cognitive ability was assessed using a composite of processing speed, social information processing, and cognitive control. This composite was found to significantly explain emotional difficulties [38]. Future studies should obtain assessments of a wide variety of cognitive constructs (e.g., attention, executive functioning, processing speed, IQ) to further determine what skills within the cognitive deficits seen in NF-1 are the most strongly related to social and emotional difficulties. Identification of specific cognitive deficits associated with social and emotional deficits can then lead to targeted interventions.

Interestingly, cognitive functioning was correlated with disease severity, indicating that lower IQ scores were found for children with symptoms of more severe disease. Perhaps the impact of cognitive functioning found in previous studies is directly related to physical disease and only indirectly related to quality of life and functional disability. Future research should examine the complexities of these potential relationships.

Historically, research on global outcomes in pediatrics has focused predominantly on HRQOL. However, recent work [21, 40] has called for the addition of a specific FD measure in conjunction with assessing global quality of life. Our findings bolster this recommendation by showing that while HRQOL was predicted by emotional functioning, it was a measure of FD that showed the effects of physical symptoms. NF-1 is a complex disease with the potential to impact myriad domains of functioning. The results of this study reflect the importance of assessing a wide variety of potential disease effects and their impact on adolescent physical functioning and quality of life.

## 5. Limitations

There are several limitations in the current study. This study did not employ an experimental design or a comparison group, which limits our ability to make causal inferences. However, the results do add to the growing literature of HRQOL research and fill a need for more research on functional disability in youth with NF-1.

Youth in this sample were predominately Caucasian. Although NF-1 usually presents equally in all racial and ethnic groups [1], this distribution is characteristic of the sample typically seen in the specialty clinic where recruiting occurred. The current sample was also restricted in terms of disease severity. The majority of participants were within the minimal to mild range. This is not representative of the range of impairment typically seen in adolescents with NF-1, and this restricted variability may have affected study results. Although the sample was limited in terms of disease severity, the sample was found to be representative of youth with NF-1 with regard to their need for educational services [41]. Half of youth received accommodations from IEP or 504 plans and 42% were in special education classes. Recruitment in future research should aim for a wider range of symptom and severity presentations to allow for greater exploration of the relationship between severity and functioning. Future studies would also be strengthened by having a larger sample size which would improve the power of the study and the range of hypotheses that could be examined.

## 6. Conclusions and Future Directions

NF-1 is a disease that can vary considerably in its medical complications and severity expression and thus might be expected to show variability in its functional impact. The findings from the current study support the need for a broad approach to the study and treatment of the sequelae of this disease. Because NF-1 can impact physical, emotional, and cognitive functioning, it is important to assess all areas when making inferences about well-being. Relying only on HRQOL to describe the functioning of adolescents with NF-1 would miss important factors such as the impact of pain on ability to perform tasks. Emotional aspects of NF-1 may be best understood by examining a global outcome such as quality of life, but to fully understand the impact of this disease the incorporation of FD is necessary. Future work can build upon these findings by examining how difficulties in physical, emotional, and cognitive domains interact to impact well-being and disability. Predictors of well-being and FD should also be examined across developmental stages and severity levels for individuals with NF-1. Finally, we hope that this work will help professionals working with children having NF-1 to understand the implications this complicated disease may have on both well-being and physical functioning to continue to improve the quality of care and support they can provide.

## Figures and Tables

**Table 1:** Descriptive information.

Scale	Minimum	Maximum	Mean	SD
Child HRQOL total score	38.04	96.74	78.34	13.98
Parent HRQOL total score	30.43	100.00	70.78	17.88
Child HRQOL physical score	46.88	100.00	82.74	13.92
Parent HRQOL physical score	34.38	100.00	80.29	16.93
Child HRQOL psychosocial score	23.33	95.00	75.99	17.63
Parent HRWOL psychosocial score	25.00	100.00	65.71	21.06
Functional disability child total score	0.00	26.00	4.62	5.30
Functional disability parent total score	0.00	25.00	3.42	6.56
BASC Child Emotional Symptoms Index	35.00	71.00	47.50	9.88
BASC parent Behavioral Symptoms Index	39.00	96.00	57.23	15.95
KBIT IQ composite	69.00	122.00	97.62	14.39
Total days of pain	0	14	4.13	3.83

**Table 2:** Bivariate correlations.

	1	2	3	4	5	6	7	8	9	10	11
(1) Gender		.06	.14	−.11	−.04	.13	−.07	−.02	.09	**−.03**	**.17**
(2) Age			.07	.12	−.29	−.02	.17	−.20	−.26	.39*	.40**
(3) Disease severity				−.28	−.45**	.14	.07	−.11	−.06	.11	.30
(4) Days of pain					.09	.31*	.16	−.17	−.33*	.21	.23
(5) IQ						0.00	−.14	−.07	.12	−.13	−.16
(6) Child FDI							.10	−.32*	.04	−.08	.22
(7) Parent FDI								−.10	−.47**	.43**	.20
(8) Child total HRQOL									.17	−.17	−.39**
(9) Parent total HRQOL										−.64**	−.26
(10) Parent BASC											.34*
(11) Child BASC											

*P < .05, **P < .01.

**Table 3:** Hierarchical multiple regressions predicting health-related quality of life.

Variable	Cumulative *R* ^2^	*F*	**β**	*R* ^2^ increment
Child HRQOL				
Step 1	.138	3.53		.138
Child age			−.37	
Step 2	.378	2.89		.240
Child BASC			−.47*	
IQ			−.10	
Days of pain			−.21	

Parent HRQOL				
Step 1	.075	1.78		.075
Child age			−.27	
Step 2	.625	7.93**		.551**
Parent BASC			−.75**	
IQ			−.13	
Days of pain			.04	

*P < .05, **P < .01.

**Table 4:** Hierarchical multiple regressions predicting functional disability.

Variable	Cumulative *R* ^2^	*F*	**β**	*R* ^2^ increment
Child FD				
Step 1	.023	.52		.023
Child age			.15	
Step 2	.545	5.69**		.522**
Child BASC			.12	
IQ			−.32	
Days of pain			.61**	

Parent FD				
Step 1	.005	.10		.005
Child age			−.07	
Step 2	.085	.44		.080
Parent BASC			.16	
IQ			.21	
Days of pain			.13	

*P < .05, **P < .01.
